# Genomic Insights of *Enterococcus faecium* UC7251, a Multi-Drug Resistant Strain From Ready-to-Eat Food, Highlight the Risk of Antimicrobial Resistance in the Food Chain

**DOI:** 10.3389/fmicb.2022.894241

**Published:** 2022-06-23

**Authors:** Mireya Viviana Belloso Daza, Giovanni Milani, Claudia Cortimiglia, Ester Pietta, Daniela Bassi, Pier Sandro Cocconcelli

**Affiliations:** Dipartimento di Scienze e Tecnologie Alimentari per una Filiera Agro-Alimentare Sostenibile (DISTAS), Università Cattolica del Sacro Cuore, Piacenza, Italy

**Keywords:** *Enterococcus faecium*, multi-drug resistant, ready-to-eat foods, genomic analysis, gene transfer

## Abstract

The presence of multi-drug resistant (MDR) bacteria in ready-to-eat foods comprises a threat for public health due to their ability to acquire and transfer antibiotic-resistant determinants that could settle in the microbiome of the human digestive tract. In this study, *Enterococcus faecium* UC7251 isolated from a fermented dry sausage was characterized phenotypically and genotypically to hold resistance to multiple antibiotics including aminoglycosides, macrolides, β-lactams, and tetracyclines. We further investigated this strain following a hybrid sequencing and assembly approach (short and long reads) and determined the presence of various mobile genetic elements (MGEs) responsible of horizontal gene transfer (HGT). On the chromosome of UC7251, we found one integrative and conjugative element (ICE) and a conjugative transposon Tn*916*-carrying tetracycline resistance. UC7251 carries two plasmids: one small plasmid harboring a rolling circle replication and one MDR megaplasmid. The latter was identified as mobilizable and containing a putative integrative and conjugative element-like region, prophage sequences, insertion sequences, heavy-metal resistance genes, and several antimicrobial resistance (AMR) genes, confirming the phenotypic resistance characteristics. The transmissibility potential of AMR markers was observed through mating experiments, where Tn*916*-carried tetracycline resistance was transferred at intra- and inter-species levels. This work highlights the significance of constant monitoring of products of animal origin, especially RTE foodstuffs, to stimulate the development of novel strategies in the race for constraining the spread of antibiotic resistance.

## Introduction

*Enterococcus faecium* is an ubiquitous species found in a large number of foods, mainly fermented products of animal origin like cheeses and fermented sausages (Braïek et al., 2018). Some strains of this species have been also recognized as probiotics conferring benefits to their hosts (Ghattargi et al., 2018). Nevertheless, in the past three decades, *E. faecium* emerged as an important nosocomial multi-drug resistant (MDR) pathogen responsible for hospital-acquired infections (Gao et al., 2018). The duality of this species has led the European Food Safety Authority (EFSA) to state a safety assessment scheme based on the absence of genetic markers generally present in the hospital-associated (HA) biotypes for those *E. faecium* strains that are intentionally introduced into the food chain (EFSA et al., 2018). Although *E. faecium* is extensively used as a probiotic and as part of the fermentation processes, it does not actually hold the qualified presumption of safety (QPS) status due to its potential pathogenicity (EFSA BIOHAZ Panel et al., 2021).

Previous studies indicated that the population structure of *E. faecium* is divided into three distinct clades: clade A1 bearing clinical isolates; clade A2 mainly represented by strains from animal and human commensals that might cause sporadic human infections, both carrying determinants for virulence and antimicrobial resistance (AMR); and clade B characterized by community-associated (CA) isolates lacking HA traits (Lebreton et al., 2013). Recently, clade B isolates were proposed to be reclassified as *Enterococcus lactis* because of their closer genomic proximity to this new species and lack of HA markers (Belloso Daza et al., 2021). The genetic transmission of HA markers among isolates, for instance, between farm animals and humans in the agricultural setting, revealed consequently the contamination of products of animal origin that affect the entire production and supply chain (Manyi-Loh et al., 2018). The rise of MDR enterococci in the food chain represents a major public health concern as they are easily disseminated through the environment (Serwecińska, 2020). Livestock animals and the farm environment exemplify an important reservoir of AMR bacteria due to the widely use of antibiotics (Koutsoumanis et al., 2021), particularly in swine for prophylactic reasons (Pholwat et al., 2020). Also, resistance to heavy metals is a matter of concern because of possible co-selection of antibiotic resistance. Specifically, resistance toward copper is common in swine-derived isolates due to the use of copper sulfate as a growth promoter in the feed for pigs (Yu et al., 2017). Enterococci-harboring MDR genes have been frequently isolated from the swine samples (Tan et al., 2018), and their diffusion arises concerns about the potential transmission to meat-based ready-to-eat (RTE) foods, which proposes a risk because of the lack of microbial inactivation prior consumption (Chajecka-Wierzchowska et al., 2019). Considering the emergence of MDR enterococci and HA isolates, the current criteria for safety assessment are represented by an MIC of ampicillin of ≤ 2 mg/L and lack of IS*16*/*esp/hyl* genes, associated with plasticity, adhesion, and carbohydrate metabolism, respectively (EFSA et al., 2018). Further information on epidemiology and the population structure can be analyzed by applying the multilocus sequence typing (MLST) scheme. Following this, *E. faecium* can be classified in different sequence types (STs), where ST17 was identified as the ancestral clone of HA isolates, forming the clonal complex 17 (CC17) (Lee et al., 2019). Nonetheless, it is crucial to understand the distribution of other putative virulence markers (PVM) involved in colonization and resistance recognized in other studies (Freitas et al., 2018; Gao et al., 2018). Horizontal gene transfer (HGT) is one of the mechanisms at the base of AMR and virulence marker dissemination among bacteria that facilitates their survival and adaptation in stressful conditions. The HGT of AMR genes between *E. faecium* and other species has been investigated mostly in clinical settings; furthermore, gene exchange in food was also demonstrated (Chajecka-Wierzchowska et al., 2019). Additionally, the transfer of resistance toward linezolid (Tyson et al., 2018), oxazolidinone (Kang et al., 2019), aminoglycosides (Kim et al., 2019), glycopeptides, erythromycin, and tetracycline (Conwell et al., 2017) has been demonstrated between food-isolated strains. The detection of AMR has also reached the retail level with the presence of AMR dissemination in RTE foods such as dairy products (Chajecka-Wierzchowska et al., 2020), salads (Zhou et al., 2020), seafood (Igbinosa and Beshiru, 2019), meat products (Chajecka-Wierzchowska et al., 2016), and pork-origin products (Kim and Koo, 2020).

Whole-genome sequencing (WGS) has facilitated the understanding of the mechanisms that support the dissemination of mobile genetic elements (MGEs) in bacteria. The aim of this study is to investigate the genomic characteristics of a vancomycin-susceptible MDR (VSE-MDR) *E. faecium* strain isolated from ready-to-eat fermented sausage and to evaluate the potential transmissibility of AMR markers through MGEs.

## Materials and Methods

### Bacterial Strain, Cultivation, and Antibiotic Susceptibility Testing

The strain UC7251 was isolated from a dry fermented Italian salami on Slanetz and Bartley Medium (Oxoid) containing 4 μg/ml ampicillin (Sigma). The strain was sub-cultivated in brain heart infusion (Oxoid) overnight at 37°C, and species-specific PCR using primers for the *ddl* gene (Supplementary Table 1S) was performed to confirm its taxonomical classification. Susceptibility to different antibiotics was determined by using the broth microdilution method according to EUCAST (EUCAST, 2022). The antimicrobial agents used were ampicillin, vancomycin, gentamycin, kanamycin, streptomycin, erythromycin, clindamycin, tylosin, tetracycline, and chloramphenicol. The antibiotics were obtained from Sigma (St. Louis, Missouri, USA). The minimum inhibitory concentrations (MICs) were compared to the breakpoints recommended by (EUCAST, 2022) (http://www.eucast.org/) and EFSA (EFSA et al., 2018).

### Heavy Metal Susceptibility Testing

Susceptibility toward copper (Cu), zinc (Zn), cadmium (Cd), and mercury (Hg) was tested, as previously described (Sharifi et al., 2015; Capps et al., 2020). Briefly, overnight cultures were spotted onto Mueller–Hinton agar (Oxoid) supplemented with different concentrations (0.05 to 40 mM) of ZnCl_2_ (Carlo Erba), HgCl_2_ (Sigma Aldrich), and CdSO_4_ (Sigma Aldrich) resuspended in distilled water, and CuSO4 (Merck Millipore) adjusted to pH 7.2 with 1 M NaOH. After 24 to 48 h of incubation at 37°C, the plates were visually inspected for bacterial growth on the spots.

### Conjugal Transfer

*In vitro* conjugation experiments were performed as described before (Cocconcelli et al., 2003). UC7251 was used as a donor strain and 29 bacterial strains as recipients (see Supplementary Table 3S). Briefly, 1 ml of a culture (OD600 = 0.8) of donor and recipient strains was passed through a 0.45-μm filter (MF-Millipore Membrane Filters, Merck). After that, the filter was placed onto non-selective agar plates favoring the growth of recipient strains and incubated at 37°C for 24 h. Conjugation with *Bacillus, Enterococcus, Listeria, Pseudomonas*, and *Staphylococcus* as recipient strains was carried out onto BHI (Oxoid), *Clostridium* on RCM (Oxoid), for lactobacilli, *Pediococcus* and *Weisella* onto MRS (Difco). After the respective incubation period, the cells were resuspended from the filter using a saline solution and were diluted in a 10-fold dilution series and enumerated by spread plating onto appropriate agar mediums. Transconjugant selection was performed using the selective conditions reported in Supplementary Table 3S. Transconjugant colonies were randomly selected and analyzed to check the presence or absence of the antibiotic resistance genes, by extracting the DNA using a microLYSIS kit (Microzone) and performing PCR with primers for tetracycline and erythromycin resistance genes (Supplementary Table 1S). The passage of potential plasmid-borne antibiotic resistance genes coding for aminoglycosides(*aad6, aph3-IIIa, aadE, satA, ant(6)-Ia)*, and lincosamides (*IsaE, LnuB*) resistance was also tested by PCR using the primers listed in Supplementary Table 1S.

### Multilocus Sequence Typing (MLST) Analysis

Allelic profiles and sequence types were derived by PubMLST (Jolley et al., 2018). The obtained ST was analyzed using Phyloviz and the goeBURST algorithm to compute a spanning forest graph to build the relatedness between isolates based on single locus variants (SLV) to identify clonal complexes (Francisco et al., 2012). Furthermore, given that the resolution of MLST is limited, core genome MLST (cgMLST) was also determined using the cgmlst.org website. This method uses an allele numbering system for a scheme of 1423 cgMLST target genes, which confers a higher level of discrimination (De Been et al., 2015).

### Detection of Markers Relevant for the Assessment of Safety and Antibiotic Resistance Determinants

The strain UC7251 was screened for the hospital-associated genetic markers IS*16, hylEfm*, and *esp* by PCR, using primers previously listed. Strains U0317 and E980 were used, respectively, as positive and negative controls. The presence of the antibiotic resistance determinants coding for the phenotypical resistances observed in UC7251 was investigated by PCR using the primers reported in Supplementary Table 1S. The complete *pbp*5 gene was amplified, sequenced, and analyzed, as described before (Pietta et al., 2014), while the amplification of *ermB, tetM, tetL, aph3-IIIa, satA, ant(6)-Ia*, and *aadE* was performed, as described elsewhere (Jacob et al., 1994; Olsvik et al., 1995; Swenson et al., 1995; Sutcliffe et al., 1996; Trzcinski et al., 2000; Ouoba et al., 2008). Here, new primers aad6_F and aad6_R for *aad6* screening, Lnu-B_F and Lnu-B_R for *Lnu(B)* screening, and IsaE_F and lsaE_R for *Isa(E)* screening were designed *de novo* using Primer3 (Untergasser et al., 2012), and the amplification reaction was run with the following conditions: initial denaturation at 95°C for 2 min; 35 cycles at 94°C for 40 s, 53°C for 45 s, and 72°C for 50 s; and extension at 72°C for 5 min.

### Genome Sequencing and Database Submission

A hybrid sequencing approach (short and long reads) was followed to complete the assembly of UC7251. Genomic DNA was extracted from the cultured bacterium with NucleoSpin Tissue (Macherey-Nagel, Germany). Short-read resequencing was performed with Illumina MiSeq, 250 paired-end after Nextera XT paired-end library preparation. Long read sequencing was performed with PacBio Sequel II SMRT sequencing. After trimming the sequences using trimgalore! (GitHub - FelixKrueger/TrimGalore), hybrid assembly was carried out using Unicycler (Wick et al., 2017). The finished genome was deposited on the NCBI under assembly accession No. ASM41165v2.

### Bioinformatics Analyses

A total of 74 *E. faecium* complete genomes, including reference strains, were selected to carry out phylogenetic and taxonomic analyses in comparison with UC7251 (Supplementary Table 2S). Assembled genomes were downloaded from the NCBI in September 2021 and were subsequently annotated using Prokka (Seemann, 2014). Annotation results were then submitted to pangenome and core genome analyses using Roary (Page et al., 2015). The phylogenetic tree was constructed using RAxML-NG, V1.0.0 (Kozlov et al., 2019), and iTOL was used to visualize and organize the tree (Letunic and Bork, 2019). The genomes were also submitted to digital DNA–DNA hybridization (dDDH) using the genome-to-genome distance calculator (GGDC) (Meier-Kolthoff et al., 2013). Average nucleotide identity (ANI) analysis was performed using fastANI (Jain et al., 2018).

*In silico* investigation of UC7251 was performed using the bioinformatics software platform Geneious Prime v. 10.1. The Basic Local Alignment Tool (BLAST) from the NCBI was used to investigate the presence and identity of different genetic markers contributing AMR, VF, and MGE. The genome was interrogated for the presence of AMR genes using the Comprehensive Antibiotic Resistance Database (CARD) (Alcock et al., 2020) and ResFinder (Bortolaia et al., 2020). Ampicillin resistance was studied by evaluating the allelic variation in the strain of interest, against the reference sequence for PBP5-S/R profiles. Virulence markers were investigated according to the latest guidelines of EFSA (EFSA et al., 2018) using manual annotation, VirulenceFinder (Joensen et al., 2014), and VFAnalyzer (Liu et al., 2018).

HGT determinants were analyzed through MobileElementFinder (Johansson et al., 2021) and IslandViewer 4 (Bertelli et al., 2017). In addition, integrative and conjugative elements were predicted using ICEberg 2.0 (Liu et al., 2019), which detects the signature sequences of the integrative modules and conjugation modules based on the profile hidden Markov models (profile HMMs). The origin of transfer site (*oriT*) was determined with OriTFinder (Li et al., 2018). Last, the genome was screened for the presence of sequences of phage origin with Prophage Hunter (Song et al., 2019) and CRISPR-Cas sites using CRISPR-CasFinder (Couvin et al., 2018).

## Results and Discussion

### Isolation and Characterization of MDR *E. faecium* UC7251 From RTE Food

In the framework of risk assessment of MDR in ready-to-eat foods, UC7251 was isolated from a dry fermented sausage at a count of 3 x 10^5^ CFU g-1 and identified as *Enterococcus faecium* by species-specific amplification of the *ddl* gene. This strain was resistant to ampicillin, streptomycin, kanamycin, erythromycin, clindamycin, tylosin, and tetracycline and presented an MIC higher than the cutoff values defined by EUCAST and EFSA (Table 1). PCR analyses, using a pool of primer pairs targeted to the most common AMR genes found in enterococci (Supplementary Table 1S), identified the genetic determinants for these resistances. *E. faecium* UC7251 was identified as a MDR strain and harbored genes coding for aminoglycoside-modifying enzymes, three genes for macrolide resistance and two genes responsible for tetracycline resistance. Moreover, the sequence of the amplicon targeted to penicillin-binding protein 5 (PBP5), involved in β-lactams resistance, demonstrated that this strain showed the *pbp5*-S1/R20 allelic profile, conferring resistance to ampicillin (Galloway-Peña et al., 2011). *E. faecium* showed to be intrinsically resistant to low levels of ampicillin through cell wall synthesis protein complex PBP; *pbp5* is part of this operon, and sequence variations allow to differentiate the two groups of *E. faecium* according to the allelic profile and expression levels (Pietta et al., 2014). Within the context of a study focusing on the detection of ampicillin-resistant *E. faecium* in ready-to-eat fermented foods, a strain that presented resistance toward ampicillin with a MIC value of 64 μg/ml and carried the hybrid allelic profile PBP5-S1/R20 is of concern for the consumers' safety. It has been demonstrated that *pbp5* may spread through horizontal gene transfer and specifically that *pbp5* of resistant isolates was located on transferable chromosomal regions, which suggested its dissemination to the environment (Morroni et al., 2019).

**Table 1:** Antimicrobial resistance genes and MIC values of strain UC7251, following the guidelines and cutoff values established by EFSA/EUCAST for the safety assessment of *E. faecium*.

**Antibiotic resistance**	**UC7251 (μg/ml)**	**EFSA Cut-off value (μg/ml)**	**EUCAST (μg/ml)**	**AMR gene**
Ampicillin	64	2	4	*pbp5-S_1_/R_20_*
Vancomycin	1	4	4	*-*
Gentamycin	32	32	32	*aac(6')-Ii*
Kanamycin	>4,096	1,024	-	*aph(3')-III*
Streptomycin	>1,024	128	128	*aad6, aadE*
Erythromycin	>512	4	4	*ermB, mrsC, sat4*
Clindamycin	>512	4	-	*ermB, InuB, IsaE*
Tylosine	>512	4	-	*ermB*
Tetracycline	128	4	4	*tetL, tetM*
Chloramphenicol	8	16	32	*-*

### Whole-Genome Sequence Analyses

UC7251 was submitted to genome sequencing following a hybrid approach using long- and short-read sequencing technology (GenBank assembly accession numbers for chromosome CP084886.1, plasmid pUC7251_1 CP084887.1, plasmid pUC7251_2 CP084888.1). The assembly of the genome of UC7251 built a total of 3 contigs, predicted as a 2.6 Mb chromosome and two plasmids, pUC7251_1 and UC7251_2 (192 kb and 1.9 kb, respectively). The presence of the two plasmids was also distinguished by total DNA extraction and pulsed-field gel electrophoresis (PFGE) (data not shown). The annotation of UC7251 resulted in 2662 coding sequences (CDS), of which 27% are hypothetical proteins and 73% have known functional assignments. It also contained genes coding for 18 rRNAs (six copies each of 23S rRNA, 16S rRNA, and 5s rRNA), 69 tRNAs, and 1 tmRNA. Compositional analysis resulted in 17 genomic islands (GIs), three active prophage sequences, and several VF and AMR genes distributed throughout the chromosome and plasmidome. Regarding mobile genetic elements, two mobile regions were predicted on the chromosome and one on pUC7251_1 (Supplementary Table 4S).

pUC7251_1 is a mobilizable megaplasmid as predicted by Plascad. According to OriTfinder, the origin of replication is 39 bp long and showed homology with *oriT_*pUB110. There are no predicted T4SS proteins and only one T4CP protein on locus tag UC7251_02595. The relaxase MobM is found on locus tag UC7251_02679. Mobilizable plasmids carry their own *oriT* and relaxase gene but lack genes required for T4SS formation and can therefore be transferred to cells that carry elements encoding a compatible T4SS (Guédon et al., 2017). This plasmid showed homology with plasmids pF88_1 (identity 83%), p17-318_1 (identity 83%), pE843-TC-299 (identity 82%), and pE843-171 (identity 80%). The first three are VSE-MDR plasmids carried by *E. faecium* strains of clade A2. These strains were isolated from environmental (pF88_1) and human samples (p17-318_1 and pE843-TC-299). The fourth plasmid, pE843-171, is carried by *Enterococcus lactis* E843, and it is characterized as VSE-MDR (Shan et al., 2022). According to these results, pUC7251_1 holds unique traits, and although the prevalence of VSE-MDR is high, none of the results on BLAST showed VSE-MDR from food origin. UC7251_2 harbors a single open reading frame that codes for a rolling circle REP (rep14a). Small plasmids of such size were also found in other *E. faecium* isolates, making it a common genomic feature.

### Phylogenomics and Population Structure Show That Foodborne UC7251 Is Neighboring HA Isolates

**Figure 1:**
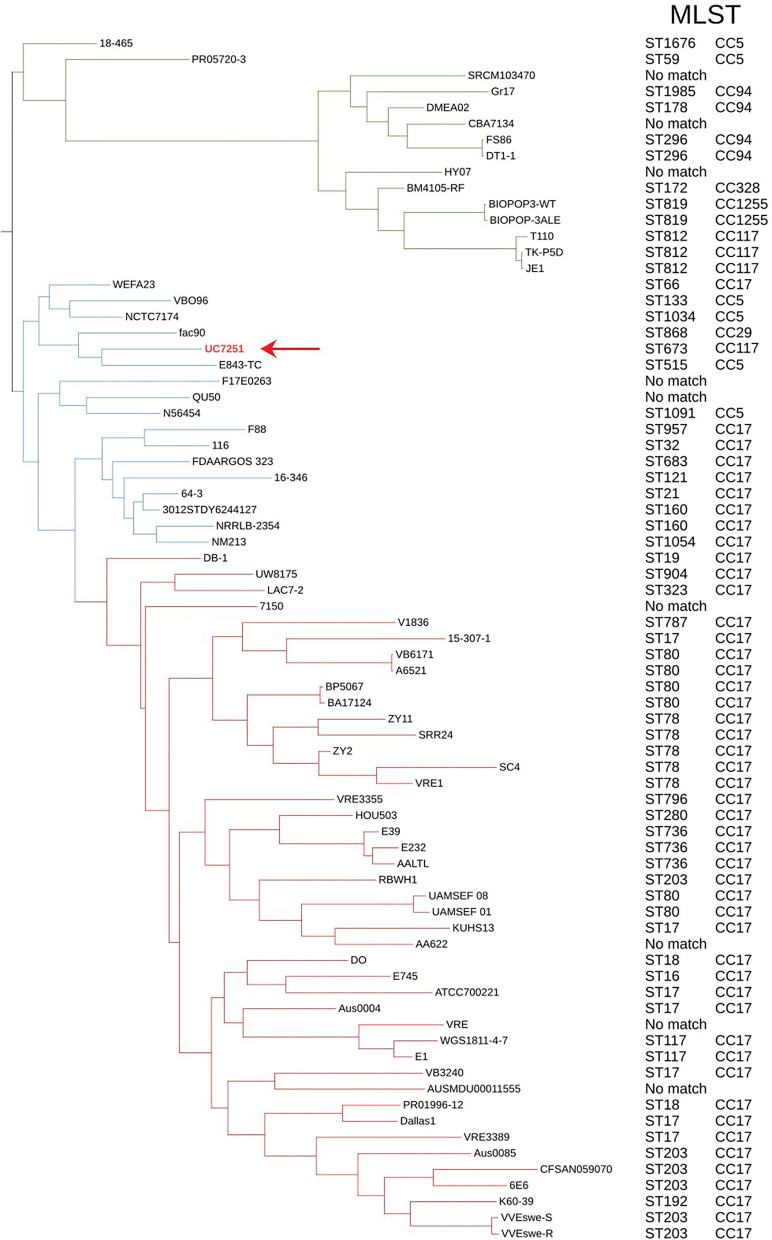
Maximum likelihood phylogenetic tree constructed using the core genome alignment of the selected 75 genomes and respective MLST and clonal complexes. *E. faecium* clade A1 strains are marked with red branches, clade A2 strains marked with blue, and clade B strains with green. Strain UC7251 (pointing arrow and text in red) is grouped among clade A2 isolates and belongs to ST673 part of CC117.

For phylogenomic evaluation, UC7251 was compared with the other selected 74 *E. faecium* genomes (Figure 1). The interrogation of the pangenome has been recently regarded as a useful tool for species delimitation based on the identification of lineage-specific gene sets (Moldovan and Gelfand, 2018). Observing the distribution of core and accessory genomes of our analysis, isolates of clade A1 and, to a smaller extent, clade A2 have a high variability in their accessory genes. A highly variable accessory genome is conferred by the fact that *E. faecium* has an open pangenome and therefore a higher genomic diversity (Lebreton et al., 2013). The adaptation of *E. faecium* to specific environmental factors, such as antimicrobial pressure, has increased the genomic diversity through horizontal gene transfer, genome rearrangement, and gene loss (Bonacina et al., 2017). Pangenome and core genome analyses uncovered an open pangenome, with a core genome consisting of 9.5% and an accessory genome of 90.5%. In this context, UC7251 contains 33 unique genes, mainly insertion sequences and hypothetical proteins located on the chromosome and on pUC7251_1. Transposases belonging to IS*3*, IS*30*, and IS*256* families were detected as unique on both pUC7251_1 and chromosome. On the chromosome, we found unique gene *arnB*, which catalyzes the conversion of UDP-4-keto-arabinose to UDP-4-amino-4-deoxy-L-arabinose. The modified arabinose is attached to lipid A and is required for resistance to polymyxin in Gram-negative bacteria (Lee and Sousa, 2014). Moreover, unique genes *epsM* and *epsL* coding for putative acetyltransferase and sugar transferase, respectively, were detected. They are involved in the production of the exopolysaccharide (EPS) component of the extracellular matrix during biofilm formation (Agius et al., 2021). Gene *cbh_2*, choloylglycine hydrolyse, catalyzes the de-conjugation of bile acids (Chand et al., 2018). In *Enterococcus*, bile salt hydrolase activity has a hypo-cholesterolemic effects on animal and human hosts, conferring probiotic properties (Singhal et al., 2019).

Furthermore, all 75 genomes were subjected to dDDH and ANI for genomic distance calculations. Although dDDH and ANI have different computational methods and species threshold values (70% for dDDH and 96% for ANI), they showed consistent results, confirming the taxonomical identification of UC7251. Digital DDH showed that values among UC7251-clade A1 strains varied from 82 to 91%, among UC7251-clade A2 strains 87-100% and among UC7251-clade B/*E. lactis* strains 64-70%. Similarly, ANI computation showed that UC7251 is closest to clade A2 strains with values between 98 and 100%, whereas comparison with genomes from the remaining two clades was lower (UC7251-clade A1: 98% and UC7251-clade B/*E. lactis*: 94%) (Supplementary Table 2S).

The population structure and location of UC7251 were also evaluated using MLST. The genome was submitted to PubMLST, and it was assigned to ST673. The latter clusters together with the clonal group of ST117, which is known to be a part of CC17 meroclone (Figure 1). Published data on PubMLST showed a unique isolate harboring ST673, which contains a strain from a non-hospitalized person collected in Spain in 2010. The MLST global scheme shows that UC7251, as other isolates from animal origin, belonged to hospital-associated clades (Gouliouris et al., 2018). Thus, *E. faecium* from CC17 have been also previously recovered from swine, poultry, and cow samples (Freitas et al., 2011; Werner et al., 2012; Getachew et al., 2013). The use of cgMLST, a clustering based on 1423 target genes of the core genome, indicated that UC7251 belonged to the unique cluster type CT745.

Subspeciation of *E. faecium* has been also studied considering the defense mechanisms against HGT, such as CRISPR-Cas systems and R-M systems (Koonin et al., 2017). CRISPR-Cas systems constitute endogenous barriers to HGT, and as a consequence, the presence of increased MGEs is associated with the complete absence or partial sequences of CRISPR-Cas systems (dos Santos et al., 2020). This has been observed in UC7251, where no complete CRISPR-Cas systems were detected. Differently, UC7251 carries, a type I R-M system with the allelic variations typical of clade A1 isolates, polymorphisms that are used for clade classification of *E. faecium* (Huo et al., 2019).

### Antimicrobial Resistance Profile and Mobilome

Several AMR genes were detected on both the chromosome and pUC7251_1 (Table 2). The intrinsic determinant coding for aminoglycoside 6′-acetyltransferase enzyme (*aac(6*′*)-II*), typical of *E. faecium* species (Chow, 2000), was found on the chromosome, together with the *liaFSR* operon, implicated in cell membrane-targeting lipopeptide antibiotic daptomycin (DAP) resistance. In previous studies, *E. faecium* isolates showed susceptibility and resistant allelic profiles of DAP (DAP-S and DAP-R, respectively) (Panesso et al., 2015); UC7251 harbors the complete *liaFSR* system with the DAP-S allelic variation. Interestingly, the occurrence of DAP resistance is inversely related to increased susceptibility to β-lactams, consistent with the ampicillin resistance in UC7251 (Diaz et al., 2014).

**Table 2:** Conjugation of tetracycline resistance between *E. faecium* UC7251 and strains from other genera.

**Donor**	**Recipient strain**	**Conjugation frequency (T/D)**	**PCR confirmation**	
			***tetM***	***tetL***
*E. faecium*	*E. faecalis* OG1rf	6.01E-03	+	-
UC7251	*L. innocua* L7	5.68E-06	+	-
	*L. monocytogenes* DSM 15675	8.38E-04	+	-
	*S. aureus* UC7180	3.78E-02	+	-
	*L. rhamnosus* UC8647	6.84E-05	+	-

**Figure 2:**
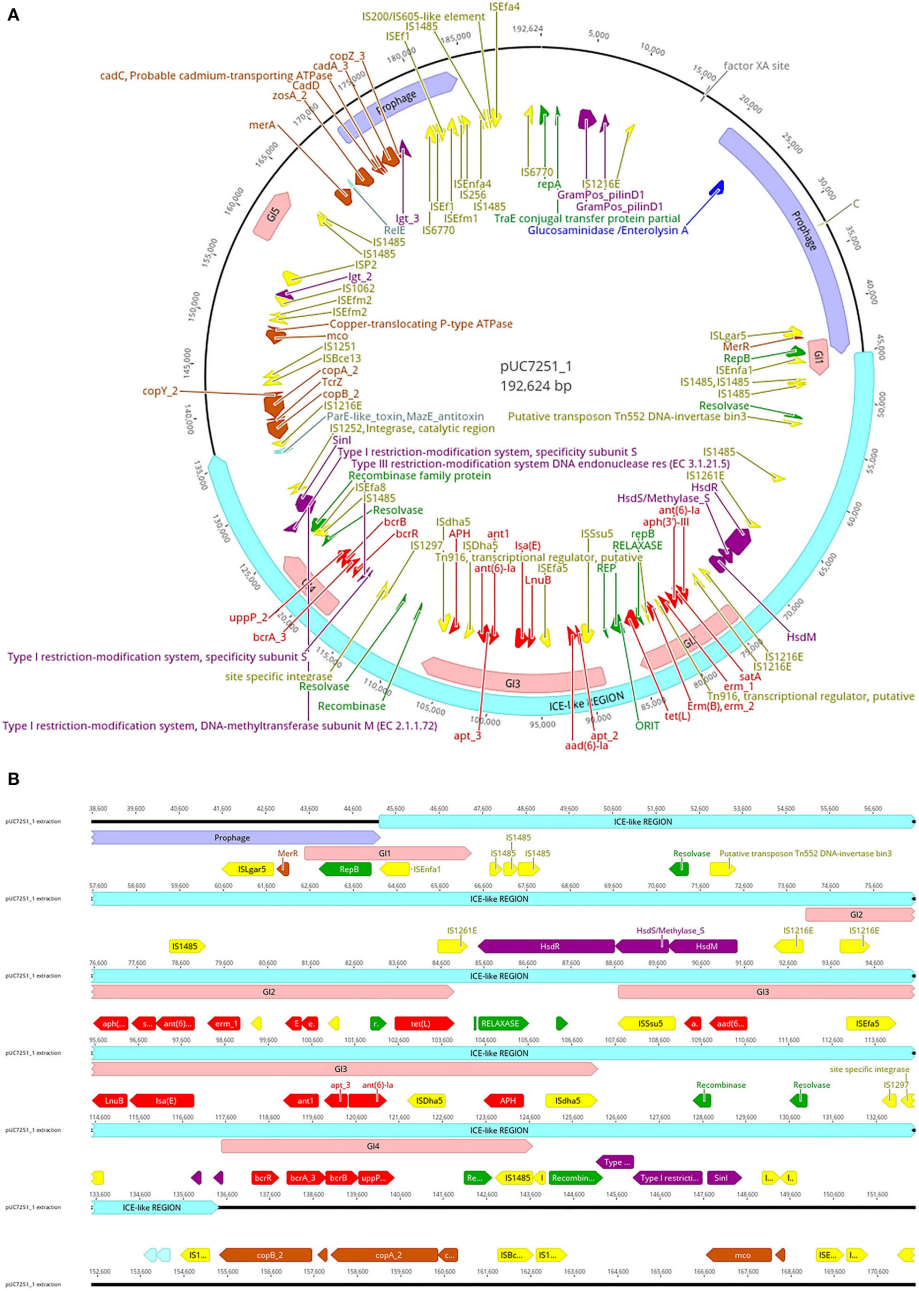
**(A)** Map of plasmid pUC7251_1 harboring one large containing prophage sequences (mauve), an integrative and conjugative-like element (cyan), five genomic islands (pink), insertion sequences (yellow), antibiotic resistance genes (red), metal resistance genes (orange), virulence factors (dark violet), replication initiation systems (green), and toxin–antitoxin systems (light blue). **(B)** Details of the ICE-like region. The genetic elements are indicated with the color code mentioned before.

Genome sequencing and assembly following a hybrid approach elucidated various details about UC7251 mobilome, crucial to understand the AMR mechanisms in this food isolated strain. A total of two mobile regions were predicted on the chromosome. Region 1 is classified as a putative integrative and mobilizable element (IME) with an insertion site and attachment sites and no detected origin of transfer (*oriT*). Proteins T2SSE, T4CP, and VirB3 are also present within this region. T4CPs are phylogenetically and structurally associated with FtsK and SpoIIIE ATPases and the ability of translocating single-stranded DNA. Furthermore, type IV secretion protein VirB3 is an inner membrane protein and requires VirB4, VirB7, and VirB8 for stabilization (Álvarez-Rodríguez et al., 2020). The IME contains several carbohydrate metabolism genes, suggesting acquired mechanisms for survival in environmental conditions. Region 2 is classified as an integrative and conjugative element (ICE), including *oriT*, insertion, and attachments sites. Additionally, it harbors T4SS machinery, integrase, relaxase, and putative transposon Tn*916*. Tn*916* is a well-described conjugative element that mediates tetracycline resistance (*tetM*) gene exchange principally among Gram-positive bacteria (Devirgiliis et al., 2009). In the same molecule, we found several inactive (score < 0.50), 2 ambiguous (score 0.5–0.79), and one putatively active (score >0.80) prophage sequences, according to the scores attributed by Prophage Hunter software. The active prophage candidate showed the closest homology to *Halocynthia* phage JM-2012 (identity of 78%). This phage is classified as a “jumbo” bacteriophage from the *Myoviridae* family, initially identified within marine *Vibrio cyclitrophicus* (Lavysh et al., 2016). Limitations of the database of the phage prediction tool may interfere with the estimation of the closest related phage. Phages from the *Myoviridae* family have been already identified in *Enterococcus* spp., making it a common feature within enterococci (Duerkop et al., 2014). Furthermore, pUC7251_1 harbors a large ICE-like gene (91 kb), with a total of four genomic islands (GIs) within (Figure 2). In detail, multiple AMR genes are found within GI2, GI3, and GI4 converting them in antibiotic resistance islands (ARI). ARI1 harbors against aminoglycoside coded by genes *aph(3')-IIIa, satA*, and *ant(6')-Ia*, found from UC7251_02667 to UC7251_02669. Interestingly, the insertion sequence IS*1216E* is found flanking this region. IS*1216* is an enterococcal IS associated with resistance toward aminoglycosides, tetracyclines, macrolides, and glycopeptides in Gram-positive bacteria (Partridge et al., 2018). IS*1216* has been identified as a vector for inter-plasmid recombination and dissemination of multi-drug elements in enterococci. Moreover, it has been found that IS*1216* is responsible for passing vancomycin resistance with the help of transposon Tn*1546* (Lin et al., 2020). Erythromycin resistance coded by *ermB* is found next to this region, and adjacently, the gene *tetL* is found next to relaxase MobM and the origin of transfer. It was reported that MobM has a dual role in autoregulation and initiation of transfer of plasmids or integrative mobilizable elements to other MGE members (Lorenzo-Díaz et al., 2018). Contiguous to this section, linezolid resistance genes *LnuB* and *IsaE* are found flanked by IS*Efa5*, which is typically found with a high copy number in *E. faecium* strains. According to a previous study, it is suggested that IS*Efa5* may be contributing significantly to the genomic flexibility of the species with evidence of frequent integration and excision events (Bayjanov et al., 2019). Additional studies have investigated that plasmids harboring linezolid resistance genes acquired from other enterococcal plasmids through MGE are associated with the MDR phenotype (Sadowy, 2018; Elghaieb et al., 2020). Conjugation of these genes was also evidenced from *Enterococcus* to *Staphylococcus*, elucidating their transmission potential (Yan et al., 2021). ARI3 carries two genes coding for aminoglycoside resistance *ant1, apt_3*, and *ant(6)-Ia*, flanked by a putative recombinase and IS*4* family transposase IS*Dha5*. IS*4* family transposases are typically found among important clinical lineages in *E. faecium* (Mikalsen et al., 2015). The presence of the complete operon for bacitracin resistance *bcrABDR* was found inside ARI4 flanked by IS*1485*. This is congruent with other studies suggesting the presence of this operon in swine isolates as it is used for prophylaxis and therapy in food animals. The plasmid co-location of this locus and other resistance gene clusters might accelerate their dissemination (Wang et al., 2015).

Regarding prophage sequences, pUC7251_1 presented two sequence fragments with high homology (score > 0.8) to known prophage sequences, classified as active. These prophages show a high identity with *Staphylococcus* phage SP beta-like and *Streptococcus* phage phiJH1301-2 prophage sequences, genetic elements that are common in clade A1 isolates (Lisotto et al., 2021). The annotated genes for both prophage sequences code mainly for transposases and integrases, as well as RelE/ParE toxin/antitoxin systems. Additionally, three ambiguous and four inactive prophage sequences were detected. Interestingly, linezolid resistance genes *IsaE, LnuB*, and bacitracin resistance operon *bcrABDR* were predicted to be within inactive prophage sequences. Recent studies have elucidated the role of phages in HGT of AMR genes as they are often carried in prophage sequences and stably inherited in the host genome-carrying antibiotic-resistant determinants (Kondo et al., 2021). Inactive or defective phages, although categorized as non-functional, still may carry out important activities and functions like transposition and excision (Mitchell, 2014).

Copper resistance operon *tcrYAZB* was also found on an inactive prophage sequence. The swine industry is well-known for using copper as a feed additive. The presence of heavy metal resistance genes is a matter of concern also because of possible co-resistance with antibiotics (EFSA FEEDAP Panel, 2016; Ahmed and Baptiste, 2018). The genetic system of this phenotypic resistance is coded by the *tcrYAZB* operon, which enhances bacterial survival and plasmid maintenance against high concentrations of this heavy metal (Rebelo et al., 2021). After genomic identification of copper resistance, we performed a susceptibility test to determine the MIC value, which resulted in 16 mM, a level typical of high copper resistance. The mobility of this operon was evidenced by flanking IS*1216E* and IS*1251*, which are highly associated within *vanA*-type plasmids (Wongnak et al., 2021). A study by Silveira et al. (2014) elucidated the presence of copper resistance genes with co-occurrence of antibiotic resistance genes. Other heavy metal resistance genes were found in pUC7251_1. Zinc chloride, oxide, or sulfate compounds are currently approved in the EU (up to 2500 ppm) and used as additives in piglet feed (Murphy et al., 2017). Similar to copper, some regulatory genes and resistance mechanisms of Cu with known links to antibiotic resistance are also zinc-responsive (Poole, 2017). UC7251 has an MIC to Zn of 16 mM and harbors gene *zosA*, observed also in *B. subtilis* for facilitating homeostasis to Zn (Argudín et al., 2019). Moreover, resistance to Cd was also determined phenotypically at 2 mM and genotypically by identifying genes *cadA, cadC*, and *cadD*. The *cad* operons are typically found in *Staphylococci* carried by MGEs like plasmids and chromosomal cassettes and co-located with antibiotic resistance genes. Specifically, *cadAC* is known for conferring high resistance levels to Cd, and it has been also reported to be present in other bacteria like *Lactococcus lactis, Streptococcus* spp., and *Listeria monocytogenes* (Argudín et al., 2019). Furthermore, resistance to Hg was conferred by two genes *merA* and *merR*. In general, resistance to Hg, is given by a set of genes clustered at the *mer* operon (*merRTPADE*) and highly linked to AMR genes and MGEs (Argudín et al., 2019). The lack of the complete operon is also confirmed by the low levels of resistance determined by MIC testing, which was of 50 μM. Genes coding for resistance to Cu, Cd, Zn, and Hg carried by plasmids has been already observed in the plasmidome of other pig isolates (Wist et al., 2020).

### Conjugation Experiments Suggest AMR Gene Transfer by Insertion Sequences

The MDR profile of UC7251 endorsed further evaluation of transmissibility of AMR genes, and we focused on tetracycline resistance coded by two genes on Tn*916* and pUC7251_1 and the plasmid encoded erythromycin resistance. This was tested through conjugation experiments where gene exchange was demonstrated at inter- and intra-generic levels (Table 2 and Supplementary Table 3S). Filter mating experiments demonstrated that tetracycline resistance was transferred from UC7251 to *E. faecalis* OG1rf, *L. innocua* L7, *L. monocytogenes* DSM 15675, *S. aureus* UC7180, and *L. rhamnosus* UC8647, with frequencies of transconjugants per donors varying from 6 x 10-3 to 5,7 x 10-6 CFU/ml. No gene transfer was observed toward Gram-negative species. The transfer of the *tetM* gene was confirmed by PCR assays, whereas *tetL* was absent in all tetracycline-positive transconjugants. The transfer of the *tetM* gene was found to be carried by chromosomal transposon Tn*916* from *E. faecalis*. This operon was predicted in chromosomal locus UC7251_02362-02376. The nucleotide identity between the 18,032-bp sequence of Tn*916* of UC7251 and *E. faecalis* (Genbank Accession No. U09422.1) sequences was of 99.97%. It has been discovered that the presence of subinhibitory concentrations of specific classes of antibiotics can trigger the mobility of Tn*916* as it has a broad inducibility of antibiotic resistance genes, implying that the dissemination of resistance genes is not necessarily linked to their selective pressure (Scornec et al., 2017).

No gene transfer for the genes coding for erythromycin resistance was observed, consistently with the characteristics of pUC7251_1, a mobilizable but non-conjugative plasmid lacking the complete conjugation apparatus.

### Virulence Markers in the UC7251 Genome Show a Collection of Colonization Facilitators

The complete assembly and annotation of UC7251 genome allowed investigation of the presence of putative virulence markers (Table 3). Adherence is an essential step in bacterial pathogenesis, required for colonization and attachment, and it is therefore considered a type of virulence marker. When scrutinizing the genome of UC7251, several microbial surface components, recognizing adhesive matrix molecules (MSCRAMMs) including LPXTG family cell wall-anchored surface proteins as well as fimbriae proteins such as pili, were identified. It is important to denote the presence of genes *acm* (cell wall-anchored collagen adhesin) and *scm* (second collagen adhesin). These proteins enhance initial adherence *in vivo* and interact with extracellular matrix components. Other genes associated with adhesion, *efaA* (*E. faecium* surface protein) and *sagA* (secreted antigen A), were detected. A novel class of cell surface proteins coded by WxL operon, found in clade A *E. faecium* isolates, with a functional role in virulence associated with endocarditis pathogenesis and bile salt resistance, was previously investigated (Galloway-Peña et al., 2015). The coding genes *swpA* (small WxL protein A), *swpB* (small WxL protein B), and *swpC* (small WxL protein C) were found in UC7251. Additionally, *malR*, a maltose-binding transcriptional regulator that increases biofilm production in the presence of this specific carbohydrate, was detected. Pili associated proteins, previously described as pilin gene clusters (PGC-1, PGC-2, PGC-3, PGC-4) (Freitas et al., 2018) were identified. PGC-1 is composed of the genes *fms20* and *fms21*; both are present along with a sortase A. This loci/operon is located between UC7251_02853 and UC7251_02588 in pUC7251_1. In addition, PGC-3 was found with 100% of nucleotide identity containing the endocarditis and biofilm-associated pili genes *ebpA, ebpB*, and *ebpC* accompanied by *srtC* (sortase) and flanked by IS*1216E*. This region is encompassed from UC7251_02583 to UC7251_02589 in the chromosome. The PGC-4 cluster is incomplete, lacking operon fms11-19-16 and PGC-2-associated genes fms14-17-13. UC7251 does not express the capsular polysaccharide, presenting the capsule operon polymorphism CPS type 1 (Hufnagel et al., 2004), and does not harbor cytolysin (Top et al., 2008) and BoNT/En toxin, a botulin-type toxin found in a single strain of *E. faecium* (Zhang et al., 2018). *E. faecium* UC7251 lacks the putative HA virulence markers as defined by EFSA (EFSA et al., 2018) and does not harbor the complete operons coding pili-associated proteins, which is typical of clade A1 isolates.

**Table 3:** Distribution of virulence factors and AMR genes including antibiotic and heavy metal resistance genes in UC7251.

**Molecule**	**Mechanism of resistance or virulence**	**Gene**	**Locus tag or position**	**Product**
Chromosome	Antibiotic	*AAC(6')-Ia*	UC7251_02097	Aminoglycoside N(6')-acetyltransferase (EC 2.3.1.82)
		*EfmM*	UC7251_02049	(rRNA) methyltransferase
		*liaFSR*	UC7251_01795-UC7251_01797	DAP
		*pbp5*	UC7251_01265	Penicillin binding protein 5
		*tet(M)*	UC7251_02367	Tetracycline resistance
	Heavy metals	*cadA_1*	UC7251_00274	Cadmium-transporting ATPase
		*cadA_2*	UC7251_00904	Cadmium, zinc and cobalt-transporting ATPase
		*copA_1*	UC7251_00909	Putative copper-importing P-type ATPase A
		*copB_1*	UC7251_00910	Copper-exporting P-type ATPase B
		*copY_1*	UC7251_00907	Transcriptional repressor CopY
		*copZ_1*	UC7251_00275	Copper chaperone CopZ
		*copZ_2*	UC7251_00908	Copper chaperone CopZ
		*cutC*	UC7251_02237	Copper homeostasis protein CutC
		*czcD*	UC7251_01786	Cadmium, cobalt and zinc/H()-K() antiporter
		*fief*	UC7251_01380	Ferrous-iron efflux pump FieF
		*ftsH*	UC7251_02411	Cell division-associated, ATP-dependent zinc metalloprotease FtsH
		*ziaA*	UC7251_01739	Zinc-transporting ATPase
		*znuA*	UC7251_02450	High-affinity zinc uptake system binding-protein ZnuA
		*znuB*	UC7251_02448	High-affinity zinc uptake system membrane protein ZnuB
		*znuC*	UC7251_02449	High-affinity zinc uptake system ATP-binding protein ZnuC
		*zosA*	UC7251_01471	Zinc-transporting ATPase
		*zupT*	UC7251_00019	Zinc transporter ZupT
		*zur*	UC7251_00846	Zinc-specific metallo-regulatory protein
	Virulence	*swpB*	UC7251_00118	Small WxL protein B
		*swpC*	UC7251_00593	Small WxL protein C
		*swpA*	UC7251_00718	Small WxL protein A
		*acm*	UC7251_02106	Cell-wall-anchored collagen adhesin, MSCRAMM
		*sagA*	UC7251_02425	Secreted antigen A
		*scm*	UC7251_02536	Second collagen adhesin, MSCRAMM
		*efaA*	UC7251_00462	Adhesion associated protein
		*BopD*	UC7251_00373	Maltose operon transcriptional repressor
		*cpsA/uppS*	UC7251_01047	Undecaprenyl diphosphate synthase uppS
		*cpsB/cdsA*	UC7251_01048	Phosphatidate cytidylyltransferase cdsA
		*fms3*	UC7251_00358	Efm surface protein 3 orf371 (PGC-4)
		*fms12*	UC7251_00496	Efm surface protein 12 orf1996 (PGC-4)
		*ebpA*	UC7251_00550	PGC-3: endocarditis- and bio- film-associated pili A (MSCRAMM)
		*epbB*	UC7251_00551	PGC-3: endocarditis- and bio- film-associated pili B (MSCRAMM)
		*ebpC*	UC7251_00552	PGC-3: endocarditis- and bio- film-associated pili C (MSCRAMM)
		*srtC*	UC7251_00553	Sortase C
		*fms6*	UC7251_00720	Efm surface protein 6 LPXTG family cell surface proteinPGC-4)
		*fms7*	UC7251_01220	Efm surface protein 7 orf2356 (PGC-4)
		*fms22*	UC7251_01278	Efm surface protein 22 orf884 (PGC-4)
		*yidC*	UC7251_00884	Inner memebrane protein translocase and chaperone
pUC7251_1	Antibiotic	*ant(6)-Ia*	UC7251_02669	Aminoglycoside 6-adenylyltransferase
		*ant1*	UC7251_02694	Streptomycin 3”-adenylyltransferase
		*ant(6)-Ia*	UC7251_02696	Aminoglycoside 6-nucleotidyltransferase
		*aph*	UC7251_02698	Aminoglycoside phosphotransferase family protein
		*Lnu(B)*	UC7251_02689	lincosamide nucleotidyltransferase
		*lsa(E)*	UC7251_02690	ABC-F type ribosomal protection protein Lsa(E)
		*tet(L)*	UC7251_02678	Tetracycline efflux MFS transporter Tet(L)
		*satA*	UC7251_02668	Streptothricin acetyltransferase A
		*erm_1*	UC7251_02671	rRNA adenine N-6-methyltransferase
		*erm_2*	UC7251_02674	rRNA adenine N-6-methyltransferase
		*aad(6)-Ia*	UC7251_02684	Aminoglycoside 6-adenylyltransferase
	Heavy metals	*copZ_3*	UC7251_02781	Copper chaperone CopZ
		*cadA*	UC7251_02780	Cadmium, zinc and cobalt-transporting ATPase
		*cadC*	UC7251_02779	Cadmium, zinc and cobalt-transporting ATPase
		*cadD*	UC7251_02778	Cadmium, zinc and cobalt-transporting ATPase
		*copA_2*	UC7251_02740	Copper-exporting P-type ATPase
		*copB_2*	UC7251_02739	Copper-exporting P-type ATPase B
		*copY_2*	UC7251_02742	Transcriptional repressor CopY
		*mco*	UC7251_02750	Multicopper oxidase mco
		*merA*	UC7251_02772	Mercuric reductase
		*merR1*	UC7251_02771	Mercuric resistance operon regulatory protein
		*TcrZ*	UC7251_02740	Copper chaperone
		*zosA*	UC7251_02776	Zinc-transporting ATPase
	Virulence	*lgt*	UC7251_02756, UC7251_02782	Surface protein anchor
		*fms20*	UC7251_02583-UC7251_02588	PGC-1: Surface protein 20
		*fms21 or pilA*	UC7251_02583-UC7251_02588	PGC-1: Surface protein 21

## Conclusion

The presence of multi-drug resistant strains in ready-to-eat fermented food represents a risk of public health for the spread of AMR determinants in the food chain and in the gut microbiota of consumers. *In silico* bioinformatics evaluations derived from genomic data permitted to accurately assess the safety of UC7251, a strain of *E. faecium* clade A2 which does not carry virulence factors typical of HA strains but presents the co-location of several antimicrobial resistance genes with heavy metal resistances on the mobilizable plasmid pUC7251_1 and the conjugative transposon Tn*916*. This work emphasizes the importance of a surveillance for the presence of AMR bacteria in food, with particular attention to fermented RTE foods. Moreover, the presence of MDR strains carrying mobile AMR genetic elements incites the development of innovative strategies for the mitigation of the risk related to antimicrobial resistance diffusion in food.

## Data Availability Statement

The datasets presented in this study can be found in online repositories. The names of the repository/repositories and accession number(s) can be found in the article/Supplementary Material.

## Author Contributions

MB: methodology, formal analysis, investigation, resources, and writing—original draft preparation. GM: methodology, investigation, and writing—original draft preparation. EP: methodology, investigation, resources, and writing—original draft preparation. CC: writing—review and editing. DB: writing—review and editing, visualization, and supervision. PC: conceptualization, writing—review and editing, validation, visualization, supervision, and project administration. All authors contributed to the article and approved the submitted version.

## Funding

This work was partially funded by the PRIMA programme, under BioProMedFood project (Reference Number: 2019-SECTION2-4; CUP: J34I19004820005). The PRIMA programme was supported by the European Union H2020 programme and innovation programme.

## Conflict of Interest

The authors declare that the research was conducted in the absence of any commercial or financial relationships that could be construed as a potential conflict of interest.

## Publisher's Note

All claims expressed in this article are solely those of the authors and do not necessarily represent those of their affiliated organizations, or those of the publisher, the editors and the reviewers. Any product that may be evaluated in this article, or claim that may be made by its manufacturer, is not guaranteed or endorsed by the publisher.

## References

[B1] Agius J. E. Phalen D. N. Rose K. Eden J. S. (2021). Genomic insights into the pathogenicity of a novel biofilm-forming enterococcus sp. bacteria (enterococcus lacertideformus) identified in reptiles. Front. Microbiol. 12, 389. 10.3389/fmicb.2021.635208 33737921 PMC7960928

[B2] Ahmed M. O. Baptiste K. E. (2018). Vancomycin-resistant enterococci: a review of antimicrobial resistance mechanisms and perspectives of human and animal health. microb. Drug Resist. 24, 590–606. 10.1089/mdr.2017.0147 29058560

[B3] Alcock B. P. Raphenya A. R. Lau T. T. Y. Tsang K. K. Bouchard M. Edalatmand A.. (2020). CARD 2020: Antibiotic resistome surveillance with the comprehensive antibiotic resistance database. Nucleic Acids Res. 48, D517–D525. 10.1093/nar/gkz935 31665441 PMC7145624

[B4] Álvarez-Rodríguez I. Arana L. Ugarte-Uribe B. Gómez-Rubio E. Martín-Santamaría S. Garbisu C.. (2020). Type IV coupling proteins as potential targets to control the dissemination of antibiotic resistance. Front. Mol. Biosci. 7, 201. 10.3389/fmolb.2020.00201 32903459 PMC7434980

[B5] Argudín M. A. Hoefer A. Butaye P. (2019). Heavy metal resistance in bacteria from animals. Res. Vet. Sci. 122, 132–147. 10.1016/j.rvsc.2018.11.007 30502728

[B6] Bayjanov J. R. Baan J. Rogers M. R. C. Troelstra A. Willems R. J. L. van Schaik W. (2019). Enterococcus faecium genome dynamics during long-term asymptomatic patient gut colonization. Microb. Genomics. 5. 10.1099/mgen.0.000277 31166888 PMC6700664

[B7] Belloso Daza M. V. Cortimiglia C. Bassi D. Cocconcelli P. S. (2021). Genome-based studies indicate that the Enterococcus faecium Clade B strains belong to Enterococcus lactis species and lack of the hospital infection associated markers. Int. J. Syst. Evol. Microbiol. 71. 10.1099/ijsem.0.004948 34402778

[B8] Bertelli C. Laird M. R. Williams K. P. Lau B. Y. Hoad G. Winsor G. L.. (2017). IslandViewer 4: expanded prediction of genomic islands for larger-scale datasets. Nucleic Acids Res. 45, W30. 10.1093/nar/gkx343 28472413 PMC5570257

[B9] Bonacina J. Suárez N. Hormigo R. Fadda S. Lechner M. Saavedra L. (2017). A genomic view of food-related and probiotic Enterococcus strains. DNA Res. An Int. J. Rapid Publ. Reports Genes Genomes. 24, 11. 10.1093/dnares/dsw043 27773878 PMC5381348

[B10] Bortolaia V. Kaas R. S. Ruppe E. Roberts M. C. Schwarz S. Cattoir V.. (2020). ResFinder 4.0 for predictions of phenotypes from genotypes. J. Antimicrob. Chemother. 75, 3491–3500. 10.1093/jac/dkaa345 32780112 PMC7662176

[B11] Braïek O. Morandi S. Cremonesi P. Smaoui S. Hani K. Ghrairi T. (2018). Biotechnological potential, probiotic and safety properties of newly isolated enterocin-producing Enterococcus lactis strains. LWT - Food Sci. Technol. 92, 361–370. 10.1016/j.lwt.2018.02.045

[B12] Capps K. M. Amachawadi R. G. Menegat M. B. Woodworth J. C. Perryman K. Tokach M. D.. (2020). Impact of added copper, alone or in combination with chlortetracycline, on growth performance and antimicrobial resistance of fecal enterococci of weaned piglets. J. Anim. Sci. 98, 1–11. 10.1093/jas/skaa003 31950170 PMC7072034

[B13] Chajecka-Wierzchowska W. Zadernowska A. García-Solache M. (2020). Ready-to-eat dairy products as a source of multidrug-resistant Enterococcus strains: Phenotypic and genotypic characteristics. J. Dairy Sci. 103, 4068–4077. 10.3168/jds.2019-17395 32197843

[B14] Chajecka-Wierzchowska W. Zadernowska A. Łaniewska-Trokenheim Ł. (2016). Diversity of antibiotic resistance Genes in *Enterococcus* Strains Isolated from ready-to-eat meat products. J. Food Sci. 81, M2799–M2807. 10.1111/1750-3841.13523 27780292

[B15] Chajecka-Wierzchowska W. Zadernowska A. Zarzecka U. Zakrzewski A. Gajewska J. (2019). Enterococci from ready-to-eat food - horizontal gene transfer of antibiotic resistance genes and genotypic characterization by PCR melting profile. J. Sci. Food Agric. 99, 1172–1179. 10.1002/jsfa.9285 30047163

[B16] Chand D. Panigrahi P. Varshney N. Ramasamy S. Suresh C. G. (2018). Structure and function of a highly active Bile Salt Hydrolase (BSH) from Enterococcus faecalis and post-translational processing of BSH enzymes. Biochim. Biophys. Acta - Proteins Proteomics. 507–518. 10.1016/j.bbapap.2018.01.003 29325872

[B17] Chow J. W. (2000). Aminoglycoside resistance in enterococci. Clin. Infect. Dis. 31, 586–589. 10.1086/313949 10987725

[B18] Cocconcelli P. S. Cattivelli D. Gazzola S. (2003). Gene transfer of vancomycin and tetracycline resistances among Enterococcus faecalis during cheese and sausage fermentations. Int. J. Food Microbiol. 315–323. 10.1016/S0168-1605(03)00194-6 14597004

[B19] Conwell M. Daniels V. Naughton P. J. Dooley J. S. G. G. (2017). Interspecies transfer of vancomycin, erythromycin and tetracycline resistance among Enterococcus species recovered from agrarian sources. BMC Microbiol. 171, 1–8. 10.1186/s12866-017-0928-3 28100194 PMC5241992

[B20] Couvin D. Bernheim A. Toffano-Nioche C. Touchon M. Michalik J. Néron B.. (2018). CRISPRCasFinder, an update of CRISRFinder, includes a portable version, enhanced performance and integrates search for Cas proteins. Nucleic Acids Res. 46, W246–W251. 10.1093/nar/gky425 29790974 PMC6030898

[B21] De Been M. Pinholt M. Top J. Bletz S. Mellmann A. Van Schaik W.. (2015). Core genome multilocus sequence typing scheme for high-resolution typing of enterococcus faecium. J. Clin. Microbiol. 53, 3788–3797. 10.1128/JCM.01946-15 26400782 PMC4652124

[B22] Devirgiliis C. Coppola D. Barile S. Colonna B. Perozzi G. (2009). Characterization of the Tn916 conjugative transposon in a food-borne strain of Lactobacillus paracasei. Appl. Environ. Microbiol. 75, 3866–3871. 10.1128/AEM.00589-09 19395574 PMC2698359

[B23] Diaz L. Tran T. T. Munita J. M. Miller W. R. Rincon S. Carvajal L. P.. (2014). Whole-genome analyses of enterococcus faecium isolates with diverse daptomycin MICs. antimicrob. Agents Chemother. 58, 4527. 10.1128/AAC.02686-14 24867964 PMC4136017

[B24] dos Santos B. A. de Oliveira J. da S. Parmanhani-da-Silva B. M. Ribeiro R. L. Teixeira L. M.. (2020). CRISPR elements and their association with antimicrobial resistance and virulence genes among vancomycin-resistant and vancomycin-susceptible enterococci recovered from human and food sources. Infect. Genet. Evol. 80, 104183. 10.1016/j.meegid.2020.104183 31923727

[B25] Duerkop B. A. Palmer K. L. Horsburgh M. J. (2014). Enterococcal Bacteriophages and Genome Defense, Enterococci: From Commensals to Leading Causes of Drug Resistant Infection. Boston, MA: Massachusetts Eye and Ear Infirmary. 24649501

[B26] EFSA BIOHAZ Panel Koutsoumanis K. Allende A. Alvarez-Ordonez A. Bolton D. Bover-Cid S.. (2021). The List of QPS Status Recommended Biological Agents for Safety Risk Assessments Carried out by EFSA.

[B27] EFSA FEEDAP Panel (2016). Revision of the currently authorised maximum copper content in complete feed. EFSA J. 14. 10.2903/j.efsa.2016.4563

[B28] EFSA Rychen G. Aquilina G. Azimonti G. Bampidis V. Bastos M. de L.. (2018). Guidance on the characterisation of microorganisms used as feed additives or as production organisms. EFSA J. 16. 10.2903/j.efsa.2018.5206 32625840 PMC7009341

[B29] Elghaieb H. Tedim A. P. Abbassi M. S. Novais C. Duarte B. Hassen A.. (2020). From farm to fork: identical clones and Tn6674-like elements in linezolid-resistant Enterococcus faecalis from food-producing animals and retail meat. *J. Antimicrob*. Chemother. 75, 30–35. 10.1093/jac/dkz419 31605129

[B30] EUCAST (2022). Break point tables for interpretation of MICs and zone diameters. Version 12.0, 2022. Available online at: http://www.eucast.org. (accessed March 09, 2022).

[B31] Francisco A. P. Vaz C. Monteiro P. T. Melo-Cristino J. Ramirez M. Carriço J. A. (2012). PHYLOViZ: Phylogenetic inference and data visualization for sequence based typing methods. BMC Bioinformatics. 13. 10.1186/1471-2105-13-87 22568821 PMC3403920

[B32] Freitas A. R. Coque T. M. Novais C. Hammerum A. M. Lester C. H. Zervos M. J.. (2011). Human and swine hosts share vancomycin-resistant Enterococcus faecium CC17 and CC5 and Enterococcus faecalis CC2 clonal clusters harboring Tn1546 on indistinguishable plasmids †. J. Clin. Microbiol. 49, 925–931. 10.1128/JCM.01750-10 21227995 PMC3067689

[B33] Freitas A. R. Tedim A. P. Novais C. Coque T. M. Peixe L. (2018). Distribution of putative virulence markers in Enterococcus faecium: towards a safety profile review. J. Antimicrob. Chemother. 73, 306–319. 10.1093/jac/dkx387 29149293

[B34] Galloway-Peña J. R. Liang X. Singh K. V. Yadav P. Chang C. La Rosa S. L.. (2015). The identification and functional characterization of WxL proteins from Enterococcus faecium reveal surface proteins involved in extracellular matrix interactions. J. Bacteriol. 197, 882–892. 10.1128/JB.02288-14 25512313 PMC4325096

[B35] Galloway-Peña J. R. Rice L. B. Murray B. E. (2011). Analysis of PBP5 of early U.S. isolates of Enterococcus faecium: Sequence variation alone does not explain increasing ampicillin resistance over time. Antimicrob. Agents Chemother. 55, 3272–3277. 10.1128/AAC.00099-11 21576454 PMC3122385

[B36] Gao W. Howden B. P. Stinear T. P. (2018). Evolution of virulence in Enterococcus faecium, a hospital-adapted opportunistic pathogen. Curr. Opin. Microbiol. 41, 76–82. 10.1016/j.mib.2017.11.030 29227922

[B37] Getachew Y. Hassan L. Zakaria Z. Abdul Aziz S. (2013). Genetic variability of vancomycin-resistant enterococcus faecium and enterococcus faecalis isolates from humans, chickens, and pigs in malaysia. Appl. Environ. Microbiol. 79, 4528–4533. 10.1128/AEM.00650-13 23666337 PMC3719509

[B38] Ghattargi V. C. Gaikwad M. A. Meti B. S. Nimonkar Y. S. Dixit K. Prakash O.. (2018). Comparative genome analysis reveals key genetic factors associated with probiotic property in Enterococcus faecium strains. BMC Genomics. 19, 1–16. 10.1186/s12864-018-5043-9 30180794 PMC6122445

[B39] Gouliouris T. Raven K. E. Ludden C. Blane B. Corander J. Horner C. S.. (2018). Genomic surveillance of enterococcus faecium reveals limited sharing of strains and resistance genes between livestock and humans in the United Kingdom. MBio. 9. 10.1128/mBio.01780-18 30401778 PMC6222123

[B40] Guédon G. Libante V. Coluzzi C. Payot S. Leblond-Bourget N. (2017). The obscure world of integrative and mobilizable elements, highly widespread elements that pirate bacterial conjugative systems. Genes (Basel). 8. 10.3390/genes8110337 29165361 PMC5704250

[B41] Hufnagel M. Hancock L. E. Koch S. Theilacker C. Gilmore M. S. Huebner J. (2004). Serological and genetic diversity of capsular polysaccharides in Enterococcus faecalis. J. Clin. Microbiol. 42, 2548. 10.1128/JCM.42.6.2548-2557.2004 15184433 PMC427887

[B42] Huo W. Adams H. M. Trejo C. Badia R. Palmer K. L. (2019). A type I restriction-modification system associated with enterococcus faecium subspecies separation. Appl. Environ. Microbiol. 85. 10.1128/AEM.02174-18 30389763 PMC6328761

[B43] Igbinosa E. O. Beshiru A. (2019). Antimicrobial resistance, virulence determinants, and biofilm formation of enterococcus species from ready-to-eat seafood. Front. Microbiol. 10. 10.3389/fmicb.2019.00728 31057497 PMC6482160

[B44] Jacob J. Evers S. Bischoff K. Carlier C. Courvalin P. (1994). Characterization of the sat4 gene encoding a streptothricin acetyltransferase in Campylobacter coli BE/G4. FEMS Microbiol. Lett. 120, 13–17. 10.1016/0378-1097(94)00168-5 8056285

[B45] Jain C. Rodriguez-R L. M Phillippy A. M. Konstantinidis K. T. Aluru S. (2018). High throughput ANI analysis of 90K prokaryotic genomes reveals clear species boundaries. Nat. Commun. 9, 1–8. 10.1038/s41467-018-07641-9 30504855 PMC6269478

[B46] Joensen K. G. Scheutz F. Lund O. Hasman H. Kaas R. S. Nielsen E. M.. (2014). Real-time whole-genome sequencing for routine typing, surveillance, and outbreak detection of verotoxigenic Escherichia coli. J. Clin. Microbiol. 52, 1501–1510. 10.1128/JCM.03617-13 24574290 PMC3993690

[B47] Johansson M. H. K. K. Bortolaia V. Tansirichaiya S. Aarestrup F. M. Roberts A. P. Petersen T. N. (2021). Detection of mobile genetic elements associated with antibiotic resistance in Salmonella enterica using a newly developed web tool: MobileElementFinder. J. Antimicrob. Chemother. 76, 101–109. 10.1093/jac/dkaa390 33009809 PMC7729385

[B48] Jolley K. A. Bray J. E. Maiden M. C. J. (2018). Open-access bacterial population genomics: BIGSdb software, the PubMLST.org website and their applications [version 1; referees: 2 approved]. *Wellcome Open Res*. 3. 10.12688/wellcomeopenres.14826.1 PMC6192448 30345391

[B49] Kang Z.-Z. Lei C.-W. Kong L.-H. Wang Y.-L. Ye X.-L. Ma B.-H.. (2019). Detection of transferable oxazolidinone resistance determinants in Enterococcus faecalis and Enterococcus faecium of swine origin in Sichuan Province, China. J. Glob. Antimicrob. Resist. 19, 333–337. 10.1016/j.jgar.2019.05.021 31136832

[B50] Kim H. J. Koo M. (2020). Diversity of enterococcus faecium in processed pork meat products in Korea. Foods. 9, 1–14. 10.3390/foods9091283 32932635 PMC7555021

[B51] Kim Y. Bin S. K. W. Son S. H. Noh E. B. Lee Y. J. (2019). Genetic characterization of high-level aminoglycoside-resistant Enterococcus faecalis and Enterococcus faecium isolated from retail chicken meat. Poult. Sci. 98, 5981–5988. 10.3382/ps/pez403 31298294

[B52] Kondo K. Kawano M. Sugai M. (2021). Distribution of antimicrobial resistance and virulence genes within the prophage-associated regions in nosocomial pathogens. mSphere. 6. 10.1128/mSphere.00452-21 34232073 PMC8386436

[B53] Koonin E. V. Makarova K. S. Wolf Y. I. (2017). Evolutionary genomics of defense systems in archaea and bacteria. Annu. Rev. Microbiol. 71, 233. 10.1146/annurev-micro-090816-093830 28657885 PMC5898197

[B54] Koutsoumanis K. Allende A. Álvarez-Ordóñez A. Bolton D. Bover-Cid S. Chemaly M.. (2021). Role played by the environment in the emergence and spread of antimicrobial resistance (AMR) through the food chain. EFSA J. 19. 10.2903/j.efsa.2021.6651 34178158 PMC8210462

[B55] Kozlov A. M. Darriba D. Flouri T. Morel B. Stamatakis A. (2019). RAxML-NG: a fast, scalable and user-friendly tool for maximum likelihood phylogenetic inference. Bioinformatics. 35, 4453–4455. 10.1093/bioinformatics/btz305 31070718 PMC6821337

[B56] Lavysh D. Sokolova M. Minakhin L. Yakunina M. Artamonova T. Kozyavkin S.. (2016). The genome of AR9, a giant transducing Bacillus phage encoding two multisubunit RNA polymerases. Virology. 495, 185–196. 10.1016/j.virol.2016.04.030 27236306 PMC13189113

[B57] Lebreton F. van Schaik W. McGuire A. M. Godfrey P. Griggs A. Mazumdar V.. (2013). Emergence of epidemic multidrug-resistant Enterococcus faecium from animal and commensal strains. MBio. 4. 10.1128/mBio.00534-13 23963180 PMC3747589

[B58] Lee M. Sousa M. C. (2014). Structural basis for substrate specificity in ArnB. a key enzyme in the polymyxin resistance pathway of gram-negative bacteria. Biochemistry. 53, 796–805. 10.1021/bi4015677 24460375 PMC3985747

[B59] Lee T. Pang S. Abraham S. Coombs G. W. (2019). Antimicrobial-resistant CC17 Enterococcus faecium: The past, the present and the future. J. Glob. Antimicrob. Resist. 16, 36–47. 10.1016/j.jgar.2018.08.016 30149193

[B60] Letunic I. Bork P. (2019). Interactive Tree of Life (iTOL) v4: recent updates and new developments. Nucleic Acids Res. 47, W256–W259. 10.1093/nar/gkz239 30931475 PMC6602468

[B61] Li X. Xie Y. Liu M. Tai C. Sun J. Deng Z.. (2018). OriTfinder: A web-based tool for the identification of origin of transfers in DNA sequences of bacterial mobile genetic elements. Nucleic Acids Res. 46, W229–W234. 10.1093/nar/gky352 29733379 PMC6030822

[B62] Lin Y.-T. Tseng S.-P. Hung W.-W. Chang C.-C. Chen You-Han J. ao Y.-T. Chen Yen-Hsu T.eng L.-J.. (2020). A possible role of insertion sequence IS1216V in dissemination of multidrug-resistant elements MESPM1 and MES6272-2 between Enterococcus and ST59 Staphylococcus aureus. Microorganisms. 8, 1–12. 10.3390/microorganisms8121905 33266174 PMC7760966

[B63] Lisotto P. Raangs E. C. Couto N. Rosema S. Lokate M. Zhou X.. (2021). Long-read sequencing-based in silico phage typing of vancomycin-resistant Enterococcus faecium. BMC Genomics. 22. 10.1186/s12864-021-08080-5 34688274 PMC8542323

[B64] Liu B. Zheng D. Jin Q. Chen L. Yang J. (2018). VFDB 2019: a comparative pathogenomic platform with an interactive web interface. Nucleic Acids Res. 47, 687–692. 10.1093/nar/gky1080 30395255 PMC6324032

[B65] Liu M. Li X. Xie Y. Bi D. Sun J. Li J.. (2019). ICEberg 2.0: an updated database of bacterial integrative and conjugative elements. Nucleic Acids Res. 47, D660–D665. 10.1093/nar/gky1123 30407568 PMC6323972

[B66] Lorenzo-Díaz F. Fernández-López C. Guillén-Guío B. Bravo A. Espinosa M. (2018). Relaxase MobM induces a molecular switch at its cognate origin of transfer. Front. Mol. Biosci. 5, 17. 10.3389/fmolb.2018.00017 29600250 PMC5863519

[B67] Manyi-Loh C. Mamphweli S. Meyer E. Okoh A. (2018). Antibiotic use in agriculture and its consequential resistance in environmental sources: potential public health implications. Molecules. 23, 795. 10.3390/molecules23040795 29601469 PMC6017557

[B68] Meier-Kolthoff J. P. Auch A. F. Klenk H. P. Göker M. (2013). Genome sequence-based species delimitation with confidence intervals and improved distance functions. BMC Bioinformatics. 14, 60. 10.1186/1471-2105-14-60 23432962 PMC3665452

[B69] Mikalsen T. Pedersen T. Willems R. Coque T. M. Werner G. Sadowy E.. (2015). Investigating the mobilome in clinically important lineages of enterococcus faecium and enterococcus faecalis. BMC Genomics. 16, 1–16. 10.1186/s12864-015-1407-6 25885771 PMC4438569

[B70] Mitchell S. (2014). Zombies in bacterial genomes: identification and analysis of previously virulent phage. Zombies Bact. Genomes Identif. Anal.

[B71] Moldovan M. A. Gelfand M. S. (2018). Pangenomic definition of prokaryotic species and the phylogenetic structure of Prochlorococcus spp. Front. Microbiol. 9, 428. 10.3389/fmicb.2018.00428 29593678 PMC5857598

[B72] Morroni G. Brenciani A. Litta-Mulondo A. Vignaroli C. Mangiaterra G. Fioriti S.. (2019). Characterization of a new transferable MDR plasmid carrying the pbp5 gene from a clade B commensal Enterococcus faecium. J. Antimicrob. Chemother. 74, 843–850. 10.1093/jac/dky549 30649343

[B73] Murphy D. Ricci A. Auce Z. Beechinor J. G. Bergendahl H. Breathnach R.. (2017). EMA and EFSA Joint Scientific Opinion on measures to reduce the need to use antimicrobial agents in animal husbandry in the European Union, and the resulting impacts on food safety (RONAFA). EFSA J. 15. 10.2903/j.efsa.2017.4666 32625259 PMC7010070

[B74] Olsvik B. Olsen I. Tenover F. C. (1995). Detection of tet(M) and tet(Q) using the polymerase chain reaction in bacteria isolated from patients with periodontal disease. Oral Microbiol. Immunol. 10, 87–92. 10.1111/j.1399-302X.1995.tb00124.x 7675524

[B75] Ouoba L. I. I. Lei V. Jensen L. B. (2008). Resistance of potential probiotic lactic acid bacteria and bifidobacteria of African and European origin to antimicrobials: Determination and transferability of the resistance genes to other bacteria. Int. J. Food Microbiol. 121, 217–224. 10.1016/j.ijfoodmicro.2007.11.018 18063151

[B76] Page A. J. Cummins C. A. Hunt M. Wong V. K. Reuter S. Holden M. T. G.. (2015). Roary: rapid large-scale prokaryote pan genome analysis. Bioinformatics. 31, 3691–3693. 10.1093/bioinformatics/btv421 26198102 PMC4817141

[B77] Panesso D. Reyes J. Gaston E. P. Deal M. Londoño A. Nigo M.. (2015). Deletion of liaR reverses daptomycin resistance in Enterococcus faecium independent of the genetic background. Antimicrob. Agents Chemother. 59, 7327–7334. 10.1128/AAC.01073-15 26369959 PMC4649183

[B78] Partridge S. R. Kwong S. M. Firth N. Jensen S. O. (2018). Mobile genetic elements associated with antimicrobial resistance. Clin. Microbiol. Rev. 31. 10.1128/CMR.00088-17 30068738 PMC6148190

[B79] Pholwat S. Pongpan T. Chinli R. Rogawski McQuade E. T. Thaipisuttikul I. Ratanakorn P.. (2020). Antimicrobial Resistance in Swine Fecal Specimens Across Different Farm Management Systems. Front. Microbiol. 11, 1238. 10.3389/fmicb.2020.01238 32625181 PMC7311580

[B80] Pietta E. Montealegre M. C. Roh J. H. Cocconcelli P. S. Murray B. E. (2014). Enterococcus faecium PBP5-S/R, the missing link between PBP5-S and PBP5-R. Antimicrob. Agents Chemother. 58, 6978–6981. 10.1128/AAC.03648-14 25182648 PMC4249377

[B81] Poole K. (2017). At the nexus of antibiotics and metals: the impact of Cu and Zn on antibiotic activity and resistance. Trends Microbiol. 25, 820–832. 10.1016/j.tim.2017.04.010 28526548

[B82] Rebelo A. Mourão J. Freitas A. R. Duarte B. Silveira E. Sanchez-Valenzuela A.. (2021). Diversity of metal and antibiotic resistance genes in Enterococcus spp. from the last century reflects multiple pollution and genetic exchange among phyla from overlapping ecosystems. Sci. Total Environ. 787, 147548. 10.1016/j.scitotenv.2021.147548 34000557

[B83] Sadowy E. (2018). Linezolid resistance genes and genetic elements enhancing their dissemination in enterococci and streptococci. Plasmid. 99, 89–98. 10.1016/j.plasmid.2018.09.011 30253132

[B84] Scornec H. Bellanger X. Guilloteau H. Groshenry G. Merlin C. (2017). Inducibility of Tn916 conjugative transfer in Enterococcus faecalis by subinhibitory concentrations of ribosome-targeting antibiotics. J. Antimicrob. Chemother. 72, 2722–2728. 10.1093/jac/dkx202 29091188

[B85] Seemann T. (2014). Prokka: rapid prokaryotic genome annotation. Bioinformatics. 30, 2068–2069. 10.1093/bioinformatics/btu153 24642063

[B86] Serwecińska L. (2020). Antimicrobials and antibiotic-resistant bacteria: a risk to the environment and to public health. Water. 12, 3313. 10.3390/w12123313

[B87] Shan X. Yang M. Wang N. Schwarz S. Li D. Du X.-D. (2022). Plasmid fusion and recombination events that occurred during conjugation of poxtA-carrying plasmids in Enterococci. Microbiol. Spectr. 10. 10.1128/spectrum.01505-21 35044200 PMC8768628

[B88] Sharifi Y. Abedzadeh A. Salighe A. Kalhor N. Motlagh M. K. Javadi A. (2015). Antibiotics and heavy metals resistance patterns of Enterococcus faecalis and faecium bacteria isolated from the human and the livestock sources. Environ. Heal. Eng. Manag. J. 2, 199–202.

[B89] Silveira E. Freitas A. R. Antunes P. Barros M. Campos J. Coque T. M.. (2014). Co-transfer of resistance to high concentrations of copper and first-line antibiotics among Enterococcus from different origins (humans, animals, the environment and foods) and clonal lineages. J. Antimicrob. Chemother. 69, 899–906. 10.1093/jac/dkt479 24343895

[B90] Singhal N. Maurya A. K. Mohanty S. Kumar M. Virdi J. S. (2019). Evaluation of bile salt hydrolases, cholesterol-lowering capabilities, and probiotic potential of enterococcus faecium isolated from rhizosphere. Front. Microbiol. 10, 1567. 10.3389/fmicb.2019.01567 31379762 PMC6646458

[B91] Song W. Sun H. X. Zhang C. Cheng L. Peng Y. Deng Z.. (2019). Prophage Hunter: an integrative hunting tool for active prophages. Nucleic Acids Res. 47, W74–W80. 10.1093/nar/gkz380 31114893 PMC6602508

[B92] Sutcliffe J. Grebe T. Tait-Kamradt A. Wondrack L. (1996). Detection of erythromycin-resistant determinants by PCR. Antimicrob. Agents Chemother. 40, 2562–2566. 10.1128/AAC.40.11.2562 8913465 PMC163576

[B93] Swenson J. M. Ferraro M. J. Sahm D. F. Clark N. C. Culver D. H. Tenover F. C.. (1995). Multilaboratory evaluation of screening methods for detection of high-level aminoglycoside resistance in enterococci. National Committee for Clinical Laboratory Standards Study Group on Enterococci. J. Clin. Microbiol. 33, 3008–3018. 10.1128/jcm.33.11.3008-3018.1995 8576363 PMC228624

[B94] Tan S. C. Chong C. W. Teh C. S. J. Ooi P. T. Thong K. L. (2018). Occurrence of virulent multidrug- resistant Enterococcus faecalis and Enterococcus faecium in the pigs, farmers and farm environments in Malaysia. PeerJ. 2018. 10.7717/peerj.5353 30123701 PMC6084283

[B95] Top J. Willems R. Bonten M. (2008). Emergence of CC17 *Enterococcus faecium* : from commensal to hospital-adapted pathogen. FEMS Immunol. Med. Microbiol. 52, 297–308. 10.1111/j.1574-695X.2008.00383.x 18279340

[B96] Trzcinski K. Cooper B. S. Hryniewicz W. Dowson C. G. (2000). Expression of resistance to tetracyclines in strains of methicillin-resistant Staphylococcus aureus. J. Antimicrob. Chemother. 45, 763–770. 10.1093/jac/45.6.763 10837427

[B97] Tyson G. H. Sabo J. L. Hoffmann M. Hsu C.-H. Mukherjee S. Hernandez J.. (2018). Novel linezolid resistance plasmids in Enterococcus from food animals in the USA. J. Antimicrob. Chemother. 73, 3254–3258. 10.1093/jac/dky369 30272180 PMC11555756

[B98] Untergasser A. Cutcutache I. Koressaar T. Ye J. Faircloth B. C. Remm M.. (2012). Primer3—new capabilities and interfaces. Nucleic Acids Res. 40, e115. 10.1093/nar/gks596 22730293 PMC3424584

[B99] Wang X.-M. Li X.-S. Wang Y.-B. Wei F.-S. Zhang S.-M. Shang Y.-H.. (2015). Characterization of a multidrug resistance plasmid from Enterococcus faecium that harbours a mobilized bcrABDR locus. J. Antimicrob. Chemother. 70, 609–632. 10.1093/jac/dku416 25324422

[B100] Werner G. Fleige C. Feßler A. T. Timke M. Kostrzewa M. Zischka M.. (2012). Improved identification including MALDI-TOF mass spectrometry analysis of group D streptococci from bovine mastitis and subsequent molecular characterization of corresponding Enterococcus faecalis and Enterococcus faecium isolates. Vet. Microbiol. 160, 162–169. 10.1016/j.vetmic.2012.05.019 22677481

[B101] Wick R. R. Judd L. M. Gorrie C. L. Holt K. E. (2017). Unicycler: resolving bacterial genome assemblies from short and long sequencing reads. PLOS Comput. Biol. 13, e1005595. 10.1371/journal.pcbi.1005595 28594827 PMC5481147

[B102] Wist V. Morach M. Schneeberger M. Cernela N. Stevens M. J. A. Zurfluh K.. (2020). Phenotypic and genotypic traits of vancomycin-resistant enterococci from healthy food-producing animals. Microorganisms 8. 10.3390/microorganisms8020261 32075283 PMC7074742

[B103] Wongnak K. Pattanachaiwit S. Rattanasirirat W. Limsrivanichakorn S. Kiratisin P. Assanasen S.. (2021). First characterization of Tn1546-like structures of vancomycin-resistant Enterococcus faecium Thai isolates. J. Infect. Chemother. 27, 991–998. 10.1016/j.jiac.2021.02.013 33663929

[B104] Yan X. M. Wang J. Tao X. X. Jia H. B. Meng F. L. Yang H.. (2021). A conjugative MDR pMG1-like plasmid carrying the lsa(E) Gene of enterococcus faecium with potential transmission to Staphylococcus aureus. Front. Microbiol. 12. 10.3389/fmicb.2021.667415 34149653 PMC8212935

[B105] Yu Z. Gunn L. Wall P. Fanning S. (2017). Antimicrobial resistance and its association with tolerance to heavy metals in agriculture production. Food Microbiol. 64, 23–32. 10.1016/j.fm.2016.12.009 28213031

[B106] Zhang S. Lebreton F. Mansfield M. J. Miyashita S. I. Zhang J. Schwartzman J. A.. (2018). Identification of a botulinum neurotoxin-like toxin in a commensal strain of Enterococcus faecium. Cell Host Microbe. 23, 169. 10.1016/j.chom.2017.12.018 29396040 PMC5926203

[B107] Zhou S. Y. D. Wei M. Y. Giles M. Neilson R. Zheng F. Zhang Q.. (2020). Prevalence of antibiotic resistome in ready-to-eat salad. Front. Public Heal. 8, 92. 10.3389/fpubh.2020.00092 32269985 PMC7109403

